# Humanized anti-CD123 antibody facilitates NK cell antibody-dependent cell-mediated cytotoxicity (ADCC) of Hodgkin lymphoma targets via ARF6/PLD-1

**DOI:** 10.1038/s41408-018-0168-2

**Published:** 2019-01-15

**Authors:** Daniel Ernst, Brent A. Williams, Xing-Hua Wang, Nara Yoon, Kyung-Phil Kim, Jodi Chiu, Zhi Juan Luo, Karin G. Hermans, Joerg Krueger, Armand Keating

**Affiliations:** 1 Cell Therapy Program, Princess Margaret Cancer Centre, Toronto, ON Canada; 2 Krembil Research Institute, University Health Network, Toronto, ON Canada; 3 Departamento de Hematología y Oncología, Facultad de Medicina, Pontificia Universidad Católica, Santiago, Chile; 4 Instituto de Ingeniería Biológica y Médica, Pontificia Universidad Católica, Santiago, Chile; 5 Program of Developmental & Stem Cell Biology, The Hospital for Sick Children, Toronto, ON Canada; 6 Division of Hematology/Oncology, The Hospital for Sick Children, Toronto, ON Canada

## Abstract

CD123 (IL-3Rα) is frequently expressed by malignant Hodgkin lymphoma (HL) cells. Naked monoclonal antibodies (mAb) against HL lack clinical benefit, partially due to absence of natural killer (NK) cells in the tumor microenvironment. Here we show that the combination of a fully humanized anti-CD123 mAb (CSL362) and high-affinity Fcγ-receptor NK-92 cells (haNK) effectively target and kill HL cells in vitro. First, we confirmed high expression of CD123 in 2 of the 3 HL cell lines (KM-H2 and L-428), and its absence in NK cells. Cytotoxicity of haNK cells against CD123-positive HL cells was significantly higher in the presence of CSL362. This was also shown with IL-15-activated primary NK cells, although haNK cells showed a 10.87-fold lower estimated half-maximal stimulatory effective concentration (EC_50_). CSL362 facilitated a significant increase in the expression of CD107a, intracellular IFN-γ and TNF-α and enhanced expression of *c-JUN*, *PLD-1*, and *ARF6* by NK cells. Inhibition of the ARF6–PLD-1 axis (NAV2729), but not of the MAPK pathway (U0126), completely abrogated CSL362-facilitated antibody-dependent cell-mediated cytotoxicity (ADCC) in haNK and activated primary NK cells. Our results support CD123 as an immunotherapeutic target for HL and the combination of NK cells and CSL362 as a treatment strategy for HL.

## Introduction

Despite increasing survival rates, about 15–25% of the patients with Hodgkin lymphoma (HL) die because of progressive disease^1^. Furthermore, a significant proportion of survivors experience long-term treatment-related complications, including secondary malignancies, cardiovascular diseases, and chronic fatigue^2–4^. Thus the development of novel, safe, and effective therapies are needed.

Natural killer (NK) cells are either absent or present in only very small numbers in the HL tumor microenvironment^5^. Moreover, residual peripheral blood and tissue-resident NK cells of HL patients are both quantitatively and qualitatively deficient, rendering them ineffective in killing and eliminating tumor cells^6–9^. NK cell-mediated antibody-dependent cell-mediated cytotoxicity (ADCC) is a major mechanism contributing to clinical efficacy of most monoclonal antibodies (mAbs) in cancer patients^10–12^. The lack of sufficient NK cells in the HL tumour microenvironment may explain the failure of naked mAbs targeting CD30, the most prominent tumor antigen of malignant HL cells, to achieve positive outcomes in phase I–II clinical trials^13–15^. NK cells have intrinsic antitumoral activity and a potential role in treating cancer patients^16–19^. Our group and others have shown that cellular therapy with allogeneic NK cells is feasible and safe, and clinically relevant responses can be achieved against a variety of malignancies^20–23^. A phase 1 clinical trial from our group for patients with relapsed and refractory hematological malignancies is the only study to include HL patients^23^. Patients were treated with the NK-92 line with established cytotoxicity against a variety of tumor types and was derived from a patient with aggressive NK cell non-HL^24^. Two of the 12 patients enrolled had HL, and 1 achieved an unmaintained remission of >10 years^23^. A genetically engineered version of the NK-92 cell line (haNK) was developed recently to express the high-affinity polymorphism (158V) of the IgG Fc-receptor (FcγRIIIa, CD16), with additional capacity for self-production of interleukin-2 (IL-2)^25^. In combination with the selected mAbs, the haNK cells demonstrated an ability to undergo ADCC in contrast to the parental CD16-negative NK-92 line and kill tumor cells^24^. The safety and feasibility of haNK cells to treat cancer patients is currently being studied in a phase 1 clinical trial (NCT03027128; NantKwest, Inc.).

CD123, the alpha chain of the IL-3 receptor (IL-3Rα), is normally expressed by early hematopoietic progenitors and cells of myeloid lineage. It is also an established tumor-associated antigen for acute myeloid leukemia (AML), particularly linked to leukemic stem cells^26^. In HL, CD123 is less recognized as a tumor antigen although it is expressed by most HL malignant cells and HL cell lines^27,28^. Moreover, the CD123+ HL lines respond to proliferative and survival signals from exogenous IL-3^27^. CSL362, also known as JNJ 56022473 or Talacotuzumab, is a fully humanized anti-CD123 mAb with additional affinity maturation and Fc-engineering to increase affinity^29^. Pre-clinical studies have demonstrated that CSL362 binds with high affinity to CD123-positive cells, inhibits tumor growth, and helps to eliminate the cancer cells in vivo^29–32^. Moreover, CSL362 can mediate ADCC by NK cells against AML blast cells; however, in one of the studies, this was restricted to allogeneic donor-derived NK cells^32^.

Because of the high frequency and intensity of CD123 expression in HL, we investigated the effectiveness of combining the fully humanized anti-CD123 mAb CSL362 with haNK cells to kill HL cells in vitro. We demonstrated that the combination of haNK and CSL362 was effective in killing HL cells and also showed that ADCC with CSL362 was associated with enhancement of the *ARF6*–*PLD-1* axis and greater lytic granule exocytosis of the haNK cells. These results support the further development of combining NK cellular therapy with the fully humanized anti-CD123 mAb to target HL.

## Materials and methods

### NK cells and HL cell lines

The HL cell lines KM-H2, L-428, and L-540, the multiple myeloma cell lines RPMI-8226 and U266, and the AML cell line K562 were cultured in RPMI-1640 (Sigma-Aldrich; St. Louis, MO) supplemented with 10% fetal bovine serum (Gibco; Gaitherburg, MD) at 37 °C in a humidified atmosphere with 5% CO_2_. The NK cell lines, NK-92 and haNK (kindly provided by NantKwest, Inc.), were cultured in X-VIVO 10 (Lonza; Anaheim, CA) plus 5% heat-inactivated human AB serum at 37 °C in a humidified atmosphere with 5% CO_2_. NK-92 cell culture media was supplemented with 450 U/ml of IL-2 (Proleukin; Novartis), while haNK cells were cultured without IL-2 because of self-production capacity. Primary NK cells were obtained from two different healthy donors. Peripheral blood mononuclear cells were isolated by Lymphoprep (Stemcell Technologies; Vancouver, BC) density gradient centrifugation. Subsequently, NK cells were negatively enriched using a NK Cell Isolation Kit (Stemcell Technologies) according to the manufacturer’s instruction. Isolated NK cells were incubated in AIM-V medium supplemented with 10% human AB serum (Sigma-Aldrich) and 10 ng/mL recombinant human IL-15 (Peprotech; Rocky Hill, NJ) for 2 weeks. Half volume of the medium was replaced twice a week. All experiments using cell lines were performed within 12 weeks of cell culture.

### Anti-CD123 mAb

The fully humanized IgG1 anti-CD123 mAb [CSL362 or JNJ 56022473] was kindly provided by Janssen Research and Development, LLC.

### Flow cytometry

All HL cell lines were tested for the expression of CD30 and CD123, both using flow cytometry (FC500, Beckman Coulter; Indianapolis, IN). Phycoerythrin (PE) anti-human CD30 and PerCP/Cy5.5 anti-human CD123 (Clone 6H6) were used for staining. Multiple myeloma cell lines were tested for CD38 expression (PE anti-human CD38). The NK cell lines NK-92 and haNK were tested for the expression of CD56 (fluorescein isothiocyanate (FITC) anti-human CD56), CD16 (PE anti-human CD16), and CD123 (PerCP/Cy5.5 anti-human CD123). For functional assays, NK cells were tested for the expression of the degranulation marker CD107a (FITC anti-human CD107a), intracellular cytokines interferon (IFN)-γ (PE anti-human IFN-γ) and tumor necrosis factor (TNF)-α (PE/Cy7 anti-human TNF-α). All antibodies used were purchased from BioLegend (BioLegend; San Diego, CA). For functional co-culture assays of effector and target cells, analysis of NK cells was performed by gating CD56-positive cells. Assays were performed on a Beckman Coulter FC500 Flow Cytometer and analysis was done using FlowJo X v.10.0.

### Cytotoxicity assays

NK-dependent tumor cell cytotoxicity was measured by the standard chromium release assay (CRA). Target HL cells (1 × 10^6^) were incubated with 100 µCi of radioactive chromium-51 (^51^Cr) for 120 min. After washing, target cells were re-suspended in serum-free media (AIM-V) and mixed in co-culture with effector cells in 96-well plates for 4 h at 37 °C in a humidified atmosphere with 5% CO_2_. Radioactivity was measured from the supernatants of each well using a gamma counter. The percentage of specific lysis was calculated using the formula [(experimental release − spontaneous release)/(maximum release − spontaneous release) × 100] and expressed as the mean of triplicate samples. Effector-to-target (E:T) ratio curves of 2.5:1, 5:1, 10:1, and 20:1 were tested for baseline cell cytotoxicity of NK-92 and haNK cells against HL cell lines. ADCC was evaluated using the CRA as described, in the presence of the fully humanized anti-CD123 mAb CSL362 (kindly provided by Jansen), and compared to the human IgG1κ isotype as well as the anti-human CD123 murine-Fc clone antibody (7G3). A dose range of 1 µg/mL–1 pg/mL for CSL362 was tested and used to estimate the half maximal stimulatory concentrations (EC_50_). E:T ratios used for ADCC testing were selected according to previous results for baseline cytotoxicity (5:1 and 10:1). The mitogen-activated extracellular signal-regulated kinase (MEK)/extracellular signal-regulated kinase (ERK) inhibitor U0126 (1 µM; Bio-Techne, Minneapolis, MN) and the ARF6 inhibitor NAV2729 (100 µM; Bio-Techne) were used for cytotoxicity analysis. The leukemia cell line K562 was used as a positive control for NK-dependent cytotoxicity.

### Degranulation and cytokine assays

To determine whether the change in cytotoxicity induced by CSL362 was dependent on an increase in granule exocytosis or in cytokine production, haNK cells were tested for the expression of the degranulation marker CD107a and the presence of the cytokines IFN-γ and TNF-α using flow cytometry. CD107a expression was analyzed using cell surface flow cytometry after a 4-h incubation period in the presence of the transport inhibitor Monensin (Biolegend), either for NK cells alone or in the presence of target cells (E:T of 10:1) with or without CSL362 (1 µg/mL). Results are expressed as the percentage of CD107a-positive cells to the total of CD56-positive cells. Analysis of IFN-γ and TNF-α was performed using intracellular staining after fixation and permeabilization and in the presence of Monensin and Brefeldin-A (all from Biolegend), according to the manufacturer’s instructions. Results are expressed as the relative variation of the median fluorescence intensity (MFI) to the control (NK cells alone).

### Real-time reverse-transcriptase (RT)-PCR

A quantitative expression analysis of *ARF6*, *c-JUN*, *PLD-1*, and *RAC-1* was performed using real-time RT-PCR for the haNK and primary NK cells. NK cells were co-incubated with target cells overnight (16 h) at 5:1 E:T ratio, with or without CSL362 (1 µg/mL). Cells were stained with PE anti-human CD16 antibody and NK cells were sorted using anti-PE MicroBeads (Miltenyi; Bergisch Gladbach, Germany). RNA was extracted from isolated NK cells (High Pure RNA Isolation Kit, Roche; Basel, Swizerland) and double-stranded complementary DNA (cDNA) was synthesized with approximately 50 ng of RNA using the High-Capacity cDNA RT Kit (ThermoFisher Scientific; Waltham, MA). Quantitative RT-PCR was performed on the 7900HT Fast RT-PCR System (Applied Biosystems; ThermoFisher) using FastStart Universal SYBR Green Master Mix (Roche) and the four-selected mRNA. The analysis was done using the SDS 2.3 software. Reactions were run in triplicates, and the genes of interest were normalized to glyceraldehyde-3P-dehydrogenase.

### Statistical analysis

Data were calculated as mean ± standard deviation (SD) of three replicates representative of at least two different experiments. Differences in the means were calculated using a two-tailed Student's *t* test or a two-way analysis of variance. A *p* value of ≤0.05 was considered statistically significant. Analysis was performed using the Prism software (Graphpad Prism 7.0a).

## Results

### HL cell lines express high levels of CD123

**Fig. 1:**
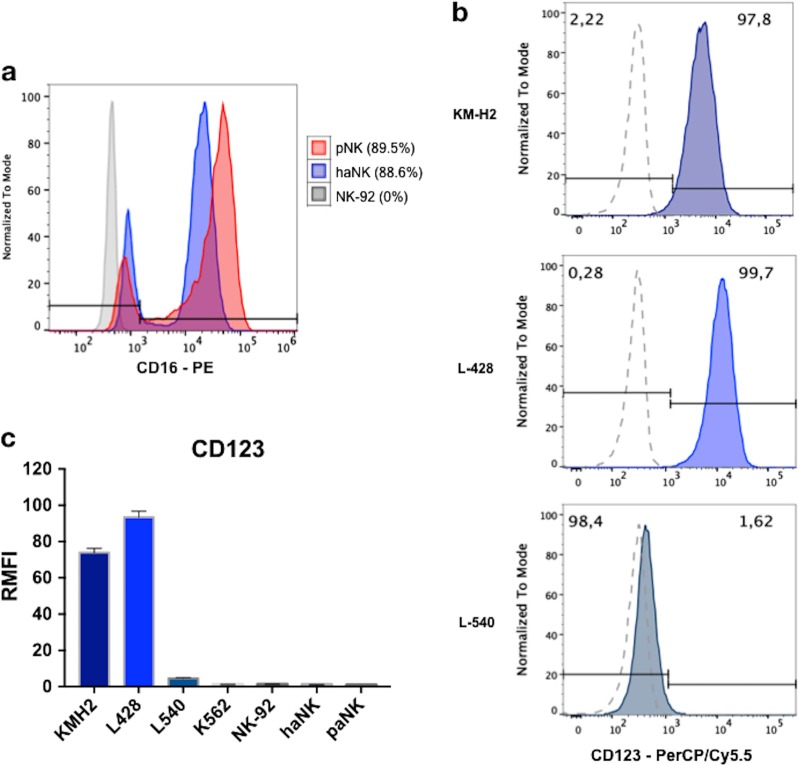
Phenotypic analysis of natural killer (NK) and Hodgkin lymphoma (HL) cells. **a** NK-92, haNK, and primary NK cells were tested for CD16 expression by flow cytometry. Gating for CD56-positive cells and CD16 expression on the each of the NK cells was compared to a murine isotype control. Additionally, HL cell lines (KM-H2, L-428, and L-540), NK cells (NK-92, haNK, and primary NK cells), and the K562 control cell line were tested for cell surface CD123. **b** Results of the HL cell lines as (%) expression. **c** Relative variation of the median fluorescence intensity (RMFI) to a murine isotype control in all cells

Phenotypic analysis was performed on all NK and target cells (HL cell lines and K562). Gating on CD56+ cells, predominant expression of CD16+ was confirmed on haNK cells (93.4%) to a level similar to primary NK cells isolated from peripheral blood. In contrast, CD16 was not expressed on NK-92 (Fig. 1a). To confirm purity of the HL cell lines and to validate the expression of CD123, cell surface expression of CD30 and CD123 were tested. As expected, CD30 was positive in all HL cell lines^33,34^. The expression of CD123 was confirmed for two of the three HL cell lines, testing positive in both KM-H2 (97.8%) and L-428 (99.7%) and negative in L-540 (1.61%) (Fig. 1b). The intensity of CD123 on both KM-H2 and L-428 was also very high, showing a relative MFI (RMFI) of 89 and 126 compared to the isotype control (Fig. 1c). NK-92, haNK and primary NK cells did not show any expression of CD123. K562, a control cell line, also tested negative for CD123 expression.

### CSL362 mediates potent and specific ADCC of NK cells against HL cells

**Fig. 2:**
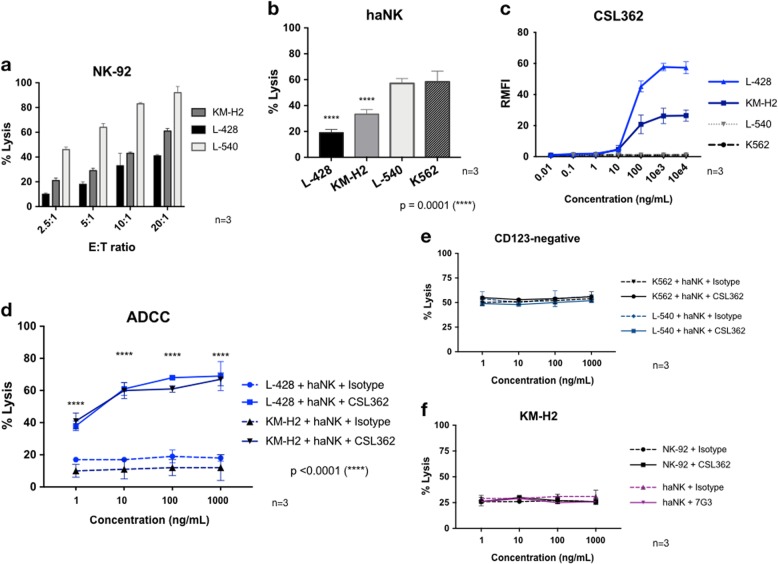
Cytotoxicity of Hodgkin lymphoma (HL) and CSL362-mediated antibody-dependent cell-mediated cytotoxicity (ADCC). **a** NK-92 dose-dependent killing efficacy of 3 HL cell lines (KM-H2, L-428, and L-540) using the 4-h standard chromium release assay (CRA). **b** At 5:1 effector-to-target (E:T) ratio, the killing efficacy of haNK cells against the three HL cell lines and the K562 control cell line is compared. **c** Analysis of CSL362-binding capacity to CD123-positive HL cells and to CD123-negative control cells. Using flow cytometry, a secondary fluorescein isothiocyanate-conjugated anti-human Fc monoclonal antibody (mAb) was added after preincubation of HL cells with increasing doses of CSL362. **d** In a 4-h CRA, CSL362-mediated ADCC was studied using haNK cells against CD123-positive HL cells at a 5:1 E:T ratio (KM-H2 and L-428). Negative ADCC controls. **e** CD123-negative target cells. **f** The murine-Fc anti-CD123 mAb (7G3) was used instead of CSL362 and NK-92 cells (CD16-negative) as effector cells

To evaluate the baseline killing efficacy of the NK-92 and haNK cells, NK cell cytotoxicity was tested against the HL cell lines KM-H2, L-428, and L-540 using the standard 4-h CRA. At increasing E:T ratios (2.5:1, 5:1, 10:1, and 20:1), an increase in dose–response was seen in all cases (Fig. 2a). At 20:1, NK-92-mediated killing of HL cell lines was 61 ± 2%, 41 ± 1%, and 92 ± 5% for KM-H2, L-428, and L-540, respectively. The efficacy of NK-92 and haNK cells against HL cell lines was compared to the NK cell target cell line K562 at E:T of 5:1 (Fig. 2b). The most sensitive cell line L-540 was killed as effectively as K562 (58 ± 8% and 57 ± 4%, respectively), but KM-H2 and L-428 were significantly less sensitive to NK cell-mediated killing (33 ± 4% and 19 ± 3%, respectively; *p* = 0.0001). Next, we confirmed the binding of the fully human anti-CD123 mAb CSL362 to CD123-positive HL cells (KM-H2 and L-428) (Fig. 2c). CSL362 binds to both cell lines at very low concentrations, having a Kd of 29.0 ng/mL (95% confidence interval (CI) 19.02–43.99) and 26.9 ng/mL (95% CI 24.52–29.45) for KM-H2 and L-428, respectively. In contrast, CSL362 did not bind to K562 (CD123-negative control) cells and also no binding to CD123-negative L-540 Hodgkin cells. Then haNK cells were tested for ADCC in the presence of increasing doses of CSL362 (1 pg/mL–1 µg/mL) against CD123-positive HL cell lines. The addition of CSL362 significantly increased the killing of the target cells in a dose-dependent fashion (Fig. 2d). No ADCC was observed when the target cells were CD123 negative (L-540 and K562) (Fig. 2e), when the CD16-negative NK-92 cell line was used as the effector cell, or when the murine Fc anti-human CD123 mAb 7G3 was used instead of CSL362 (Fig. 2f).

### The combination of haNK cells and CSL362 is active in the pg/mL range

**Fig. 3:**
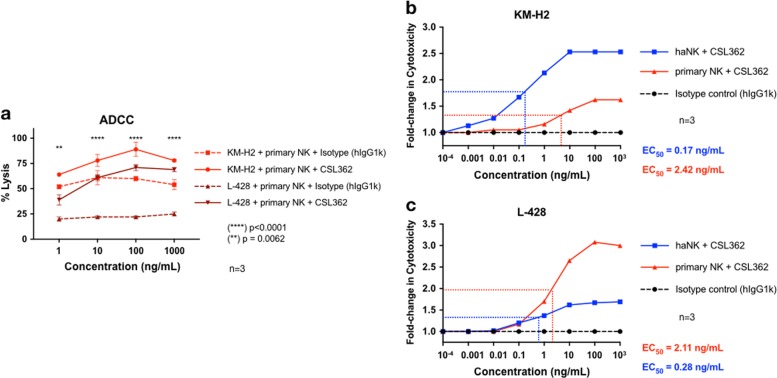
Antibody-dependent cell-mediated cytotoxicity (ADCC) of primary natural killer (NK) cells. Results of one representative donor, tested in triplicate. **a** CSL362-mediated ADCC validation using primary interleukin-15 (10 ng/mL) activated NK cells against CD123-positive HL cells (KM-H2 and L-428) in a 4-h chromium release assay; 5:1 effector-to-target ratio. Estimation of the half-maximal stimulatory effective concentration (EC_50_) of CSL362 (1 µg/mL–1 pg/mL) in combination with haNK cells or activated primary NK cells against KM-H2 (**b**) and L-428 cells (**c**)

Because haNK cells were transduced with high-affinity polymorphism (V158F) CD16, we compared the estimated half-maximal stimulatory effective concentrations (EC_50_) of CSL362 when combined with haNK or IL-15-activated primary NK cells. The activated NK cells from two different donors were also active against HL cells, and in the presence of CSL362, a significant increase in cytotoxicity against CD123-positive HL cells was observed (Fig. 3a). The combination of haNK cells and CSL362 achieved an EC_50_ of 0.17 ng/mL and 0.28 ng/mL for KM-H2 (Fig. 3b, c) and L-428, respectively. The combination of activated primary NK cells and CSL362 had EC_50_ of 2.42 ng/mL for KM-H2 and 2.11 ng/mL for L-428, which is approximately ten times lower compared with haNK cells (Fig. 3b, c).

### CSL362 induces an increase in lytic granule exocytosis and cytokine production

**Fig. 4:**
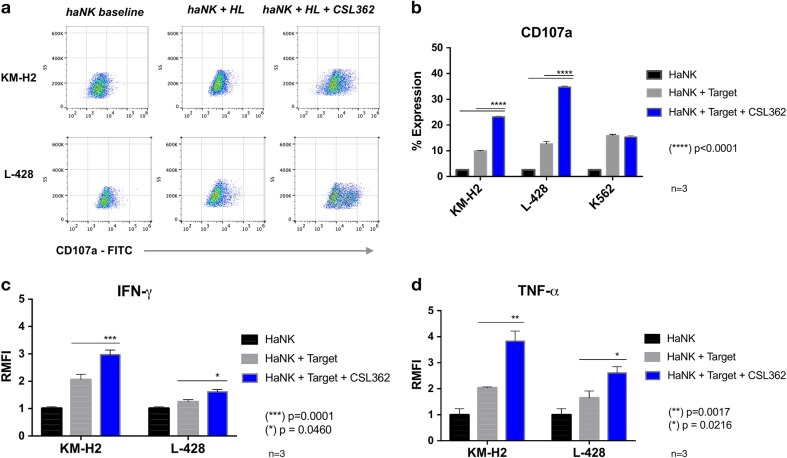
Degranulation markers and cytokines. **a** haNK cells and target cells were co-cultured at 5:1 effector-to-target (E:T) ratio for 4 h with and without CSL362 (1 µg/mL). Using flow cytometry and gating for CD56+ cells, the dot plot expression of the degranulation marker CD107a vs standard side scatter is shown. **b** Cumulative expression of CD107a compared to the CD123-negative control cell line K562. The presence of interferon-γ (**c**) and tumor necrosis factor-α (**d**) was estimated by comparing the relative variation of the median fluorescence intensity (RMFI) of haNK cells in co-culture with L-428 cells (5:1 E:T ratio) in the presence or absence of CSL362 (1 µg/mL), to that of untreated haNK cells alone

To determine the mechanisms of CSL362-mediated increase in cytotoxicity of the NK cells, we tested the expression of CD107a on the cell surface and the intracellular presence of IFN-γ and TNF-α, using flow cytometry. The analyses of CD107a, IFN-γ and TNF-α were performed after 4-h co-culture. In the presence of CSL362 (1 µg/mL), the expression of CD107a was significantly increased compared to the CD107a expression in the absence of CSL362. This was observed in both cases of co-culture with KM-H2 cells (23.13% ± 0.6 vs 9.93% ± 0.4; *p* < 0.0001) and L-428 target cells (34.73% ± 0.7 vs 12.7% ± 1.7; *p* < 0.0001) (Fig. 4a, b). In contrast, no change in the expression of CD107a was observed when the CD123-negative K562 cells was used as a target (15.90% ± 0.4 vs 15.35% ± 0.32; *p* = 0.7358). In addition, the presence of intracellular cytokines in haNK cells was increased in the presence of CSL362 (Fig. 4c, d). IFN-γ expression by haNK cells was increased by 44.17% (2.97 ± 0.28 vs 2.06 ± 0.34; *p* = 0.0001) and 29.39% (1.61 ± 0.16 vs 1.24 ± 0.15; *p* = 0.0460) against KM-H2 and L-428, respectively. Similarly, TNF-α expression was increased by 88.06% (3.84 ± 0.54 vs 2.04 ± 0.05; *p* = 0.0017) and 58.39% (2.61 ± 0.47 vs 1.65 ± 0.53; *p* = 0.0216).

### Activation of NK cells by CSL362 leads to higher levels of c-JUN and PLD-1

**Fig. 5:**
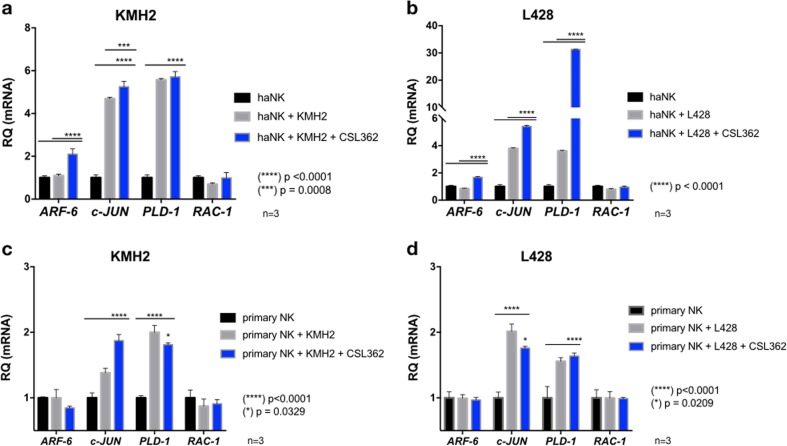
RNA expression in natural killer (NK) cells. HaNK cells were cultured for 16 h with CD123-positive Hodgkin lymphoma **a** KM-H2 and **b** L-428 cells at 5:1 effector-to-target ratio, in the presence or absence of CSL362 (1 µg/mL). Later, cells were stained for PE anti-human CD16 and sorted using anti-PE magnetic microbeads. The expression of *ARF6*, *c-JUN*, *PLD-1*, and *RAC-1* RNA was analyzed using real-time reverse-transcriptase PCR and expressed relative to haNK cells under baseline culture conditions. **c**, **d** show a similar experiment using primary NK cells activated with IL-15 (10 ng/mL) as effector cells

In order to identify the likely pathway responsible for ADCC with CSL362, we studied the changes in mRNA expression of *c-JUN*, *PLD-1*, and *RAC-1* as the downstream regulators of the NK cell degranulation pathways. Because phospholipase D1 (PLD-1) and Ras-related C3 botulinum toxin substrate 1 (RAC-1) are both controlled by ADP-ribosylation factor 6 (ARF6), we also tested the expression of *ARF6* mRNA. HaNK and activated primary NK cells were co-incubated (E:T 5:1) with KM-H2 or L-428 cells overnight in the presence or absence of CSL362 (1 µg/mL). mRNA expression was analyzed using RT-PCR and compared to resting haNK and activated primary NK cells. K562 target cells were used as controls (Supplemental Fig. 1). While *RAC-1* expression was not significantly modified, *c-JUN* and *PLD-1* were more highly expressed in haNK and activated primary cells in contact with HL cells (Fig. 5a–d). HaNK cells exposed to CSL362 had a significantly greater expression of *c-JUN* (KM-H2 and L-428; *p* = 0.0008 and *p* < 0.0001, respectively) and of *PLD-1* (L-428; *p* < 0.0001). The stimulatory effect of CSL362 was maximal for the haNK cells in co-culture with L-428, having an absolute increase of 27.7-fold (31.31 ± 0.08 vs 3.61 ± 0.04; *p* < 0.0001) in *PLD-1* expression (Fig. 5b). Moreover, *ARF6* was significantly increased in haNK cells exposed to CSL362 (*p* < 0.0001) and not after co-culture with KM-H2 and L-428 cells without CSL362. Similar findings were observed in the activated primary NK cells, with the exception of the *ARF6* increase seen in haNK cells (Fig. 5c, d).

### Inhibition of ARF6 abrogates ADCC in NK cells

**Fig. 6:**
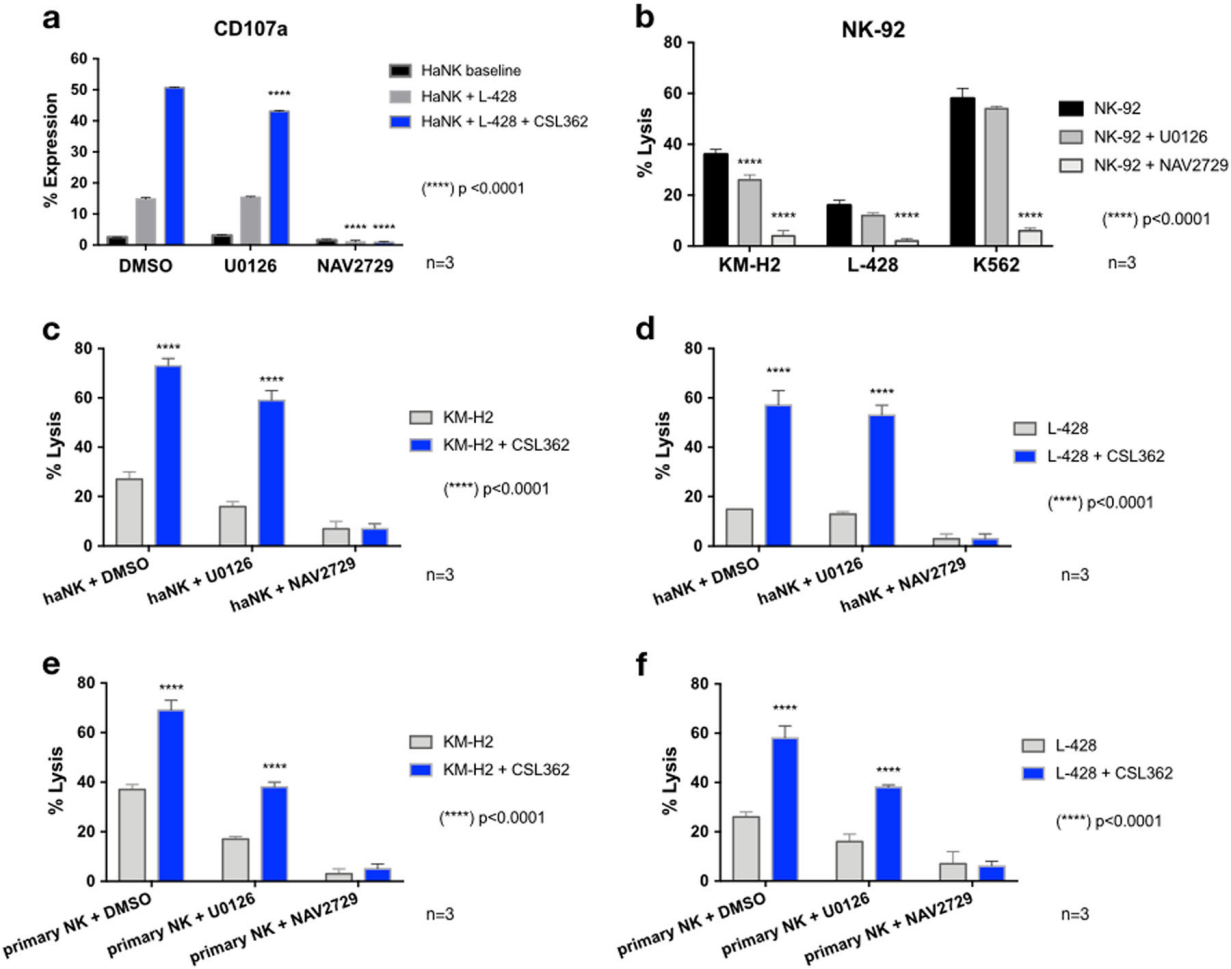
The role of mitogen-activated protein kinase (MAPK)/c-JUN and ARF6/PLD-1 in cytotoxicity and antibody-dependent cell-mediated cytotoxicity (ADCC). Natural killer (NK) cells were cultured in the presence of U0126 (1 µM), NAV2729 (100 µM), or dimethyl sulfoxide (DMSO) control (0.1%) for 24 h prior to each experiment. **a** Cumulative flow cytometric expression of CD107a in a 4-h co-culture of haNK cells and L-428 cells at 5:1 effector-to-target (E:T) ratio, in the presence or absence of CSL362 (1 µg/mL). **b** Four-h chromium release assay (CRA) of NK-92 cells vs KM-H2, L-428, and K562 cells (5:1 E:T), without CSL362. Cytotoxicity in a 4-h CRA of haNK cells against **c** KM-H2 cells and **d** L-428 cells in the presence or absence of CSL362 and similarly of activated (IL-15 at 10 ng/mL) primary NK cells against **e** KM-H2 cells and **f** L-428 cells

After the confirmation of the upregulation of the *MAPK/c*-*JUN* and the *ARF6/PLD-1* axes upon CSL362 activation of CD16, we studied their functional role in cytotoxicity and ADCC. NK-92, haNK, and activated primary NK cells were preincubated for 24 h using MEK inhibitor (U0126), ARF6 inhibitor (NAV2729), or dimethyl sulfoxide control prior to the co-culture with target cells and CSL362. Earlier, the concentration of NAV2729 was established at 100 µM, without a negative effect on the NK cells’ viability (Supplemental Fig. 2). Flow cytometric analysis on CD107a expression showed that U0126-pretreated haNK cells co-cultured with L-428 cells in the absence of CSL362 expressed similar amounts of CD107a compared to haNK cells in standard conditions but showed a significant decrease in CD107a in the presence of CSL362 (43.1 ± 0.44 vs 50.7 ± 0.3; *p* < 0.0001) (Fig. 6a). HaNK cells preincubated with NAV2729 showed a significant decrease in CD107a, both in the presence (0.80 ± 0.43 vs 50.7 ± 0.3; *p* < 0.0001) and absence (0.97 ± 0.90 vs 14.79 ± 0.88; *p* < 0.0001) of CSL362. Furthermore, CRA showed that preincubation with U0126 significantly decreased cytotoxicity of KM-H2 cells compared to NK-92 cells under standard conditions (36 ± 2 vs 26 ± 2; *p* < 0.0001) but not of L-428 or K562 (Fig. 6b). Meanwhile, NAV2729-pretreated NK-92 cells showed a significantly lower cytotoxicity of KM-H2 (36 ± 2 vs 4 ± 2; *p* < 0.0001), L-428 (16 ± 2 vs 2 ± 1; *p* < 0.0001), and K562 (58 ± 4 vs 6 ± 1; *p* < 0.0001). In the presence of CSL362, U0126-pretreated haNK cells showed a significant increase in cytotoxicity of KM-H2 (59 ± 4 vs 16 ± 2; *p* < 0.0001) and L-428 (53 ± 4 vs 13 ± 1; *p* < 0.0001), compared to U0126-pretreated haNK cells in the absence of CSL362 (Fig. 6c, d). In contrast, in both the presence and absence of CSL362, NAV2729-pretreated haNK cells showed no change in cell lysis of KM-H2 (7 ± 3 vs 7 ± 2; *p* > 0.9999) and L-428 cells (3 ± 2 vs 3 ± 2; *p* > 0.9999). Similar findings were observed when activated primary NK cells were used as effector cells (Fig. 6e, f). In the presence of CSL362, U0126-pretreated activated primary NK cells performed significantly greater cytotoxicity against KM-H2 (38 ± 2 vs 17 ± 1; *p* < 0.0001) and L-428 (38 ± 1 vs 16 ± 3; *p* < 0.0001) compared to U0126-pretreated activated primary NK cells without CSL362. Also, in the presence or absence of CSL362, NAV2729-pretreated activated primary NK cells showed similarly low cytotoxicity levels against KM-H2 (5 ± 2 vs 3 ± 2; *p* < 0.0001) and L-428 (6 ± 2 vs 7 ± 5; *p* < 0.0001).

### The role of the ARF6–PLD-1 axis in ADCC is not exclusive to HL

**Fig. 7:**
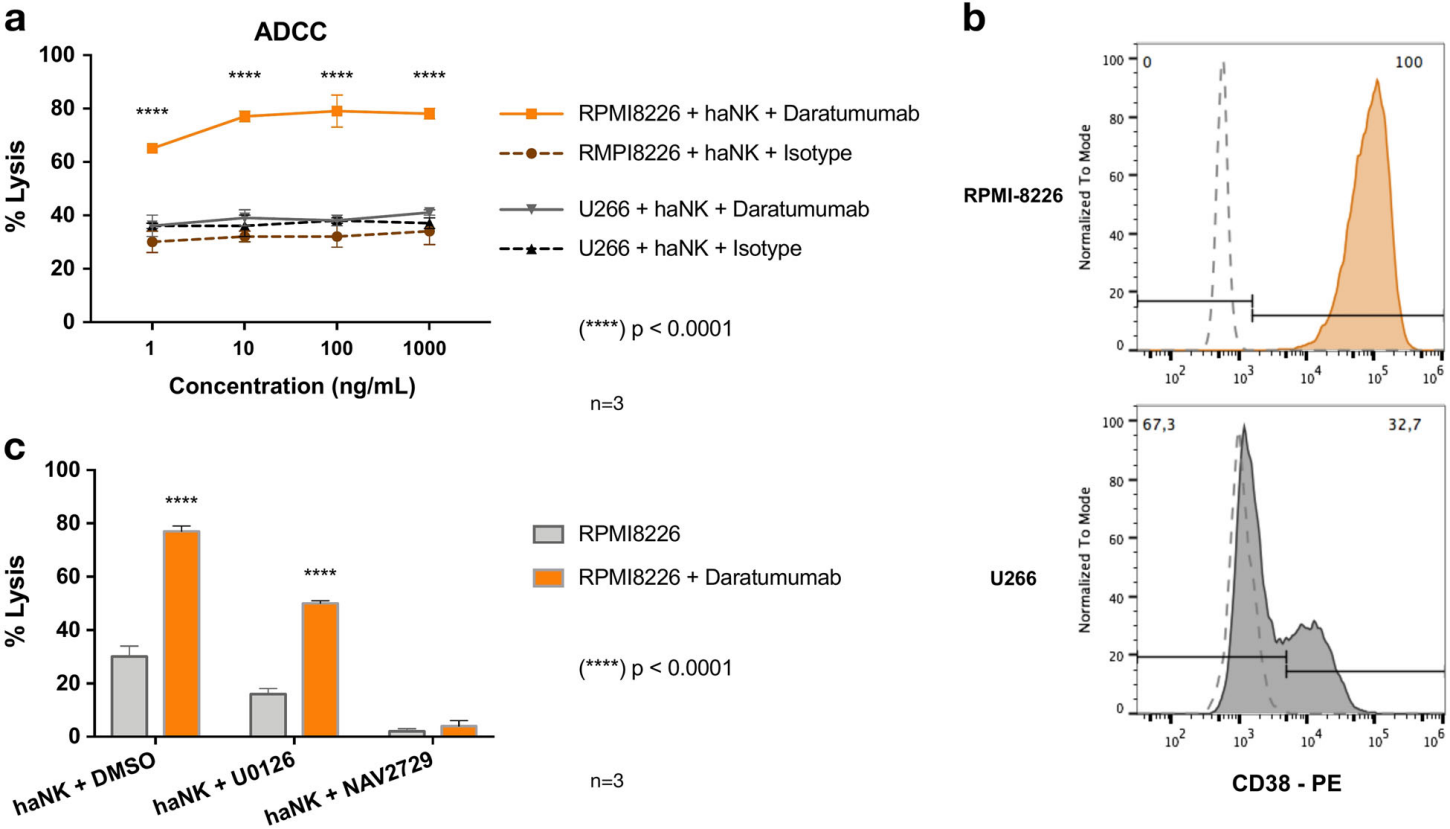
Cytotoxicity of multiple myeloma cells. **a** Daratumumab-mediated antibody-dependent cell-mediated cytotoxicity (ADCC) was evaluated using the 4-h chromium release assay of haNK cells against RPMI-8226 and U266 cells at 5:1 effector-to-target ratio. **b** Cell surface CD38 expression of RPMI-8226 and U266 cells compared to the respective murine isotype control. **c** Effect in cytotoxicity of U0126 (1 µM) and NAV2729 (100 µM) 24 h pretreatment of haNK cells vs RPMI-8226 cells, in the presence or absence of Daratumumab (1 µg/mL)

Because the ARF6–PLD-1 axis plays a major role in NK cell-mediated cytotoxicity, we evaluated its contribution using an established model of ADCC. HaNK cells were tested for cytotoxicity using the fully humanized anti-CD38 mAb Daratumumab (Darzalex^®^) against two multiple myeloma cell lines (RPMI-8226 and U266). The presence of Daratumumab was associated with a significant increase in the killing of RPMI-8226 target cells but not of U266 cells (Fig. 7a). This correlated with the level of expression of CD38, positive at 100% and 32.7% for RPMI-8226 and U266, respectively (Fig. 7b), and showing an RMFI of 169 and 2, respectively. Additionally, when haNK cells were pretreated with U0126 and NAV2729 and in the absence of Daratumumab, a significantly lower cytotoxicity was observed (30 ± 4 vs 16 ± 2 vs 2 ± 1; *p* < 0.0001) (Fig. 7c). In the presence of Daratumumab, U0126-pretreated haNK cells showed a significant increase in the killing of RPMI-8226 cells (50 ± 1 vs 16 ± 2; *p* < 0.0001), while NAV2729-pretreated haNK cells failed to mediate ADCC (4 ± 2 vs 2 ± 1; *p* = 0.6493).

## Discussion

Our study demonstrates that the combination of CD16-positive NK cells (haNK and primary NK cells) and the fully humanized anti-CD123 mAb CSL362 is highly active against CD123-expressing HL. To our knowledge, this is the first study to show the efficacy of combining NK cells with an mAb to target HL. Moreover, our present study validates the capacity of NK cells to mediate potent and specific ADCC against HL when coupled with the fully humanized anti-CD123 mAb (CSL362)^25^.

We have shown the potential utility of NK cells as a source of cellular therapy to treat HL. Baseline cytotoxicity results showed variability based on the cell line targeted (at E:T of 5:1, 19%, 33%, and 58% for L-428, KM-H2, and L-540, respectively) and may reflect the heterogeneity of patient tumors in clinical practice. Even under low E:T ratios, however, the haNK cell and CSL362 combination improves the cytotoxicity by several folds. Despite the variable sensitivity of different HL cell lines to both haNK and activated primary NK in the presence of CSL362, the absolute maximal level of cytotoxicity is similarly high. This suggests that the additional cytotoxicity of NK cells provided by the anti-CD123 mAb overcomes the inhibitory mechanisms displayed by the tumor cells^35^.

CSL362 binds to CD123 at very low concentrations and the EC_50_ is in the pg/mL range. These values are approximately 10 times higher than the combination with primary NK cells, in line with the expected contribution of the high-affinity CD16 on haNK^25^. Although primary NK cell donors were not tested for the 158V polymorphism, the differences in the EC_50_ are likely due to the low frequency of the polymorphism in the normal population^11,36^. A low EC_50_ could enable the use of lower doses of CSL362 to achieve a clinical response, compared with the higher doses of CSL362 needed without haNK. This is particularly relevant given the hematological toxicities observed with anti-CD123 therapies^37^. Moreover, haNK cells have the advantage of uniform potency, expression of the high-affinity CD16 found only a minority of the population homozygous for the FcγRIIIa (CD16) 158V polymorphism^11,36^, and are readily expandable for use as a universal and off-the-shelf source of NK cells, making the therapeutic combination of haNK cells and mAbs feasible. Additionally, limiting adverse effects, including severe cytokine release syndrome, encephalopathy, persistent B cell aplasia (in the case of anti-CD19 CAR-T cells), or death, related to other forms of cellular therapy are usually not associated with NK cells^38–41^.

The significant increase in cytotoxicity from CSL362 is mainly due to enhancement of the *ARF6*–*PLD1* axis, leading to an increase in granule exocytosis by NK cells. When exposed to HL cells, haNK cells showed increased expression of *c-JUN* and *PLD-1*, an effect further enhanced by CSL362. Activation of the *ARF6*–*PLD-1* axis appears to contribute more significantly than does the *MAPK/c-JUN* pathway in producing cytotoxic effects. Inhibition of ARF6 by NAV2729 had a significant negative impact on cytotoxicity of the NK-92/haNK cells. NAV2729-pretreated haNK did not express CD107a in response to CD123+ HL cells in the presence of CSL362 and were completely unable to mediate ADCC. In contrast, MEK/ERK inhibition by U0126 yielded only a small decrease in cytotoxicity, without reduction of CD107a expression or ADCC of haNK cells, in contact with CD123+ targets in the presence of CSL362. While activation of the MAPK pathway leads to proliferation and survival of NK cells, enhancement of cytotoxicity is due mainly to IFN-γ and TNF-α production and less from lytic granule secretion via PLA-2^42,43^. In contrast, the ARF6–PLD-1 axis regulates protein trafficking, including lytic granule exocytosis of NK cells^44,45^. ARF6 activates PLD-1 to couple CD16 activation with ADCC.

Our data also show for the first time the role of the ARF6–PLD-1 axis in NK cell-mediated ADCC of cancer cells. The roles of ARF6 and PLD-1 in ADCC appear to be similar among the NK cells we studied, including for primary NK cells. Based on similar results with an anti-CD38 antibody in myeloma and CSL362 with HL, we infer that our observation of the involvement of ARF6–PLD-1 may be generally applicable in NK-mediated ADCC. Our results also show that the activity of ARF6–PLD-1 is not exclusive to the CD16 pathway and may represent a final common pathway of the downstream activation of several activating receptors. Specifically, blocking the ARF6–PLD-1 pathway with NAV2729 inhibited baseline cytotoxicity of NK cells against tumor targets in the absence of antibody. This highlights the importance of this signalling pathway in NK cell cytotoxicity.

In conclusion, we have shown that targeting CD123 on HL cells with a humanized anti-CD123 mAb (CSL362) and NK cells capable of engaging in ADCC is highly effective in vitro. We have also demonstrated that this enhanced cytotoxicity occurs via ARF6–PLD-1 activation. Our data suggest that the combination of the high-affinity CD16+ NK line, haNK, and a humanized anti-CD123 mAb, CSL362, suggests a novel approach to investigate in the treatment of patients with relapsed/resistant CD123+ HL.

## Supplementary information

Supplemental figures

Supplemental Figure 1&2 legend
