# Supplementary figures and images for: Humanized anti-CD123 antibody facilitates NK cell antibody-dependent cell-mediated cytotoxicity (ADCC) of Hodgkin lymphoma targets via ARF6/PLD-1
Source: Blood Cancer J. 2019 Jan 15;9(2):6. doi: 10.1038/s41408-018-0168-2 (PMC6333842; doi:10.1038/s41408-018-0168-2)

# Supplemental Figure 1

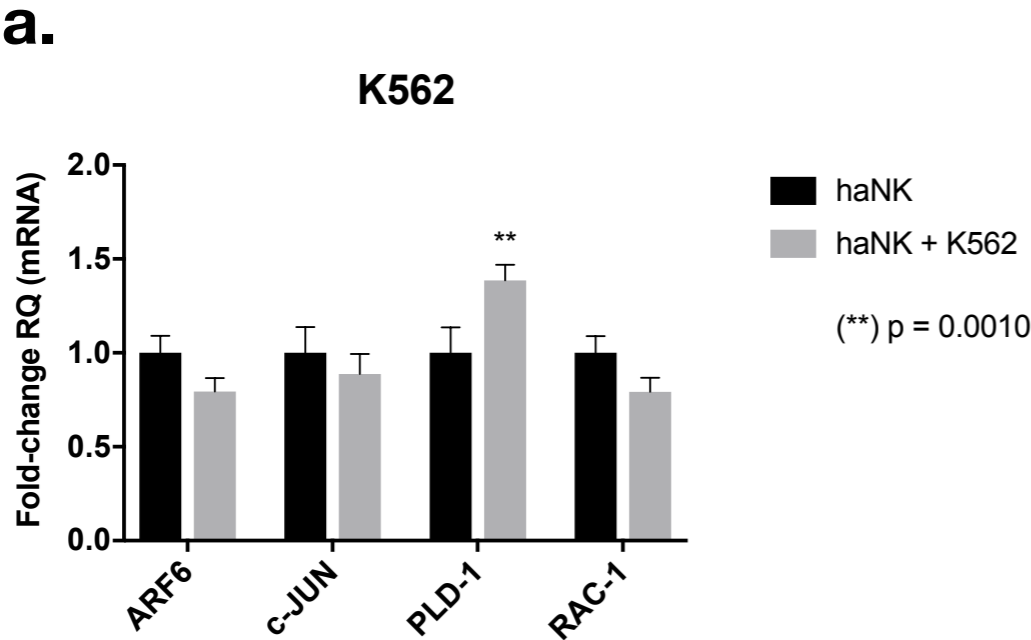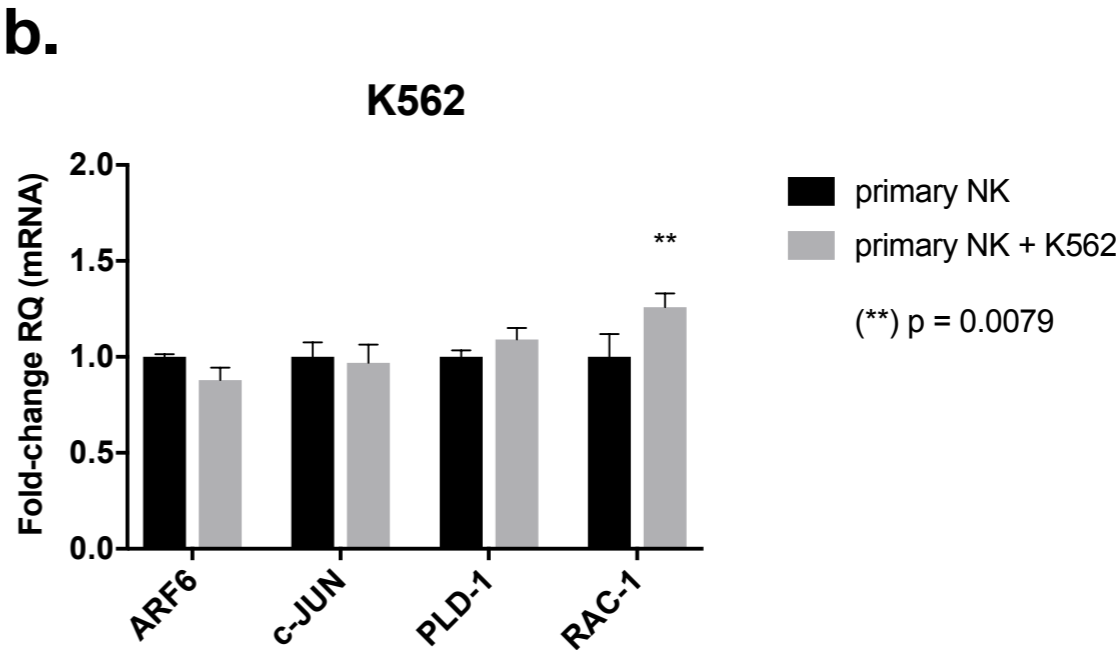

# Supplemental Figure 2

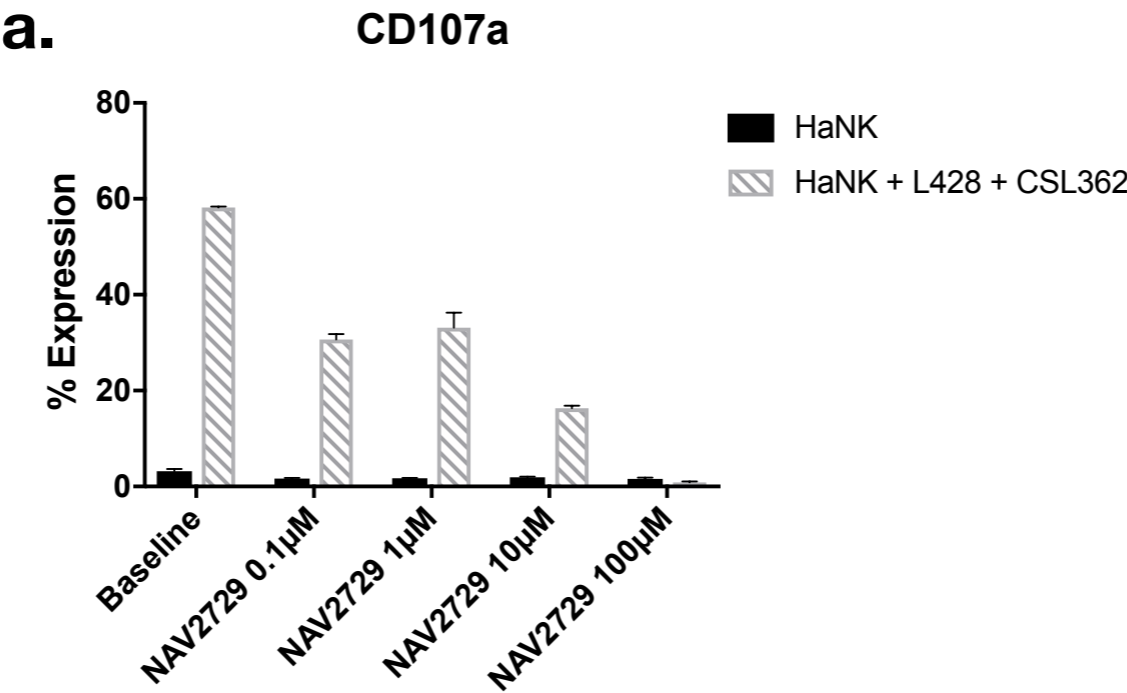

**b.**

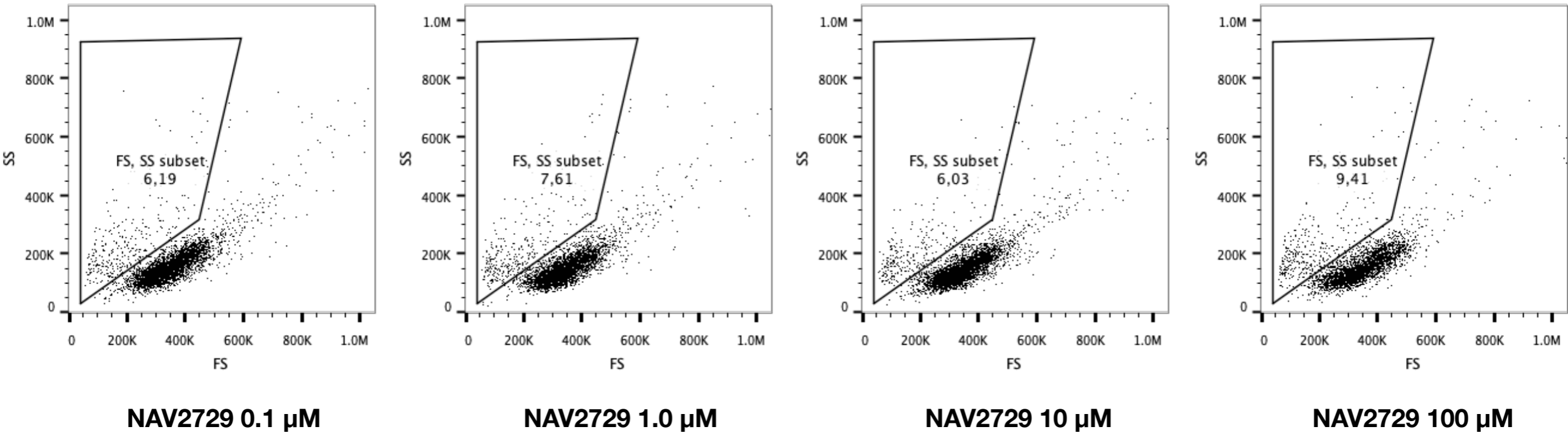

Supplement: Supplementary file 1 — Supplemental figures [file 41408_2018_168_MOESM1_ESM.pdf]
