# Supplementary material for: Humanized anti-CD123 antibody facilitates NK cell antibody-dependent cell-mediated cytotoxicity (ADCC) of Hodgkin lymphoma targets via ARF6/PLD-1
Source: Blood Cancer J. 2019 Jan 15;9(2):6. doi: 10.1038/s41408-018-0168-2 (PMC6333842; doi:10.1038/s41408-018-0168-2)
Supplement: Supplementary file 2 — Supplemental Figure 1&2 legend [file 41408_2018_168_MOESM2_ESM.docx]

**Supplemental Figure 1**: RNA expression in NK cells. CD123-negative K562 cells were cultured for 16-hours with (a.) hank cells and (b.) primary NK cells at 5:1 effector to target ratio. Later, cells were stained for PE anti-human CD16 and sorted using anti-PE magnetic microbeads. The expression of *ARF-6, c-JUN, PLD-1* and *RAC-1* RNA was analyzed using real-time reverse-transcriptase PCR and expressed relative to NK cells under baseline culture conditions.

**Supplemental Figure 2**: Effect of NAV2729 (ARF6 inhibitor) on haNK cells. NK cells were cultured in the presence of incremental doses of NAV2729 (0.1 µM, 1.0 µM, 10 µM and 100µM) or DMSO control (0.1%) for 24 hours. (a.) Cumulative flow cytometry expression of CD107a after a 4-hour co-culture of haNK cells and L-428 cells, in the presence of CSL362 (1 µg/mL), at 5:1 effector to target (E:T) ratio, or haNK cells alone. (b.) FS/SS gates show percentage of non-viable haNK cells after a 24-hour culture in the presence of increasing concentrations of NAV2729.
